# Assessing the validity of prospective hazard analysis methods: a comparison of two techniques

**DOI:** 10.1186/1472-6963-14-41

**Published:** 2014-01-27

**Authors:** Henry WW Potts, Janet E Anderson, Lacey Colligan, Paul Leach, Sheena Davis, Jon Berman

**Affiliations:** 1 Centre for Health Informatics and Multiprofessional Education, Institute of Epidemiology & Health Care, University College London, London, UK; 2 Florence Nightingale School of Nursing and Midwifery, King’s College London, London, UK; 3 Division of Neonatology, University of Virginia, Charlottesville, USA; 4 Green Street Berman Ltd., London, UK; 5 CHIME, 3rd floor, Wolfson House, 4 Stephenson Way, London NW1 2HE, UK

**Keywords:** Risk assessment, Reliability and validity, HFMEA, SWIFT, Prospective hazard analysis

## Abstract

**Background:**

Prospective Hazard Analysis techniques such as Healthcare Failure Modes and Effects Analysis (HFMEA) and Structured What If Technique (SWIFT) have the potential to increase safety by identifying risks before an adverse event occurs. Published accounts of their application in healthcare have identified benefits, but the reliability of some methods has been found to be low. The aim of this study was to examine the validity of SWIFT and HFMEA by comparing their outputs in the process of risk assessment, and comparing the results with risks identified by retrospective methods.

**Methods:**

The setting was a community-based anticoagulation clinic, in which risk assessment activities had been previously performed and were available. A SWIFT and an HFMEA workshop were conducted consecutively on the same day by experienced experts. Participants were a mixture of pharmacists, administrative staff and software developers. Both methods produced lists of risks scored according to the method’s procedure. Participants’ views about the value of the workshops were elicited with a questionnaire.

**Results:**

SWIFT identified 61 risks and HFMEA identified 72 risks. For both methods less than half the hazards were identified by the other method. There was also little overlap between the results of the workshops and risks identified by prior root cause analysis, staff interviews or clinical governance board discussions. Participants’ feedback indicated that the workshops were viewed as useful.

**Conclusions:**

Although there was limited overlap, both methods raised important hazards. Scoping the problem area had a considerable influence on the outputs. The opportunity for teams to discuss their work from a risk perspective is valuable, but these methods cannot be relied upon in isolation to provide a comprehensive description. Multiple methods for identifying hazards should be used and data from different sources should be integrated to give a comprehensive view of risk in a system.

## Background

Prospective hazard analysis (PHA) methods are a powerful way to increase understanding of risks in a system and implement changes before harm occurs [1,2]. They have a long history in high risk systems such as transportation, nuclear power and the military [3,4]. Many PHA methods have been developed, including Failure Modes and Effects Analysis (FMEA), Hazard and Operability (HAZOP), Systematic Human Error Reduction and Prediction Approach (SHERPA) and Technique for Human Error Rate Prediction (THERP) [5]. These methods share a generally similar structure: an experienced, multi-disciplinary analysis team is assembled; documentation about the system is produced, including procedure manuals, process maps or task analyses; the analysis team systematically examines the process for potential risks as guided by the method [3]. In this study, we examined the validity of two methods, Healthcare Failure Modes and Effects Analysis (HFMEA) and Structured What If Technique (SWIFT). HFMEA is a version of Failure Modes and Effects Analysis adapted for the requirements of healthcare [6] and is probably the best known prospective method in healthcare. It requires a significant investment in time and personnel for effective use and this may be a factor limiting its use. SWIFT is a structured brainstorming technique that uses guide words and prompts to identify risks [7,8]. There is no specific healthcare version, but it can be tailored by developing guide words and prompts specific to the problem being analysed [7,8]. We applied these methods in a community-based anticoagulation monitoring service and investigated whether they produced similar results.

Healthcare has been slow to adopt PHA methods, [9] notably in the UK where such methods are not mandated. Health care organisations rely to a large extent on retrospective methods, including incident reporting, root cause analysis and patient complaints, to identify risks [10]. Retrospective methods address problems after they have occurred, rather than preventing them prospectively. Kessels-Habraken and colleagues [11] have shown that prospective methods identify different risks than those identified by retrospective methods, and have provided guidance on how the results of the two different approaches can be integrated to provide a comprehensive view of risks in a system. Others have also recommended that PHA and retrospective methods should be used together to provide complementary views [12].

There are several possible explanations for the slow uptake of these methods in healthcare. First, the variety of different methods available makes it difficult to know which is the most suitable method for a particular risk assessment. Lyons [9] has provided an overview of available methods to assist the novice healthcare user. Others have also provided guidance for applying some methods in healthcare, including FMEA [4].

Second, using these methods is resource intensive, [13] especially if they are applied in the recommended form. Prospective methods are collaborative and require a multi-disciplinary analysis team to be fully effective [2]. Many methods can take several days to apply, [9] but less complex systems may require less complex methods [3]. Less intensive techniques have been developed, like the Structured What-If Technique (SWIFT), [14] that aim to achieve most of the results of other techniques in a fraction of the time, but little is known about their effectiveness.

Third, despite examples of the benefits of applying these methods, [5,15,16] there is little evidence for their reliability or validity [3,17]. Shebl and colleagues [18] tested the reliability of FMEA by having two analysis groups apply the technique to the same process. The groups mapped out the process of care in a similar way, but only a small number of risks were identified by both groups. Of those that were, severity ratings were different between the two groups. Similarly, discrepancies in HFMEA scores were found by Ashley and Armitage [19] who found differences in severity ratings determined by consensus between team members and those determined by averaging team members’ individual scores.

The potential value of structured methods is the reduction of variability that can be achieved by guiding analysts to attend to relevant information in a systematic way. However, there is little evidence available to indicate which methods might do this more effectively. Although there have been some investigations of method reliability, less is known about the validity of methods and whether different methods produce different results. Validation of PHA methods is methodologically challenging. Ideally, safety should improve after conducting the analysis and implementing interventions, but it would be difficult to isolate the effect of the analysis alone on outcome measures. A criterion measure of validity against which to compare method outcomes would also be difficult to identify as risk analysis is a subjective process and risks can never be fully known. The extent to which results produced by different methods overlap, or converge, could be a more useful measure of validity. Risk and hazard analysis techniques provide a structured process for an essentially qualitative analysis; analysts use their judgement and experience to identify potential events and ways to prevent them occurring. Some variability in the results obtained by different groups at different times in different organisations should therefore be expected, along with some overlap. In this paper, we investigate to what extent two prospective hazard analysis methods produce results that overlap with each other and with prior safety measures.

### Aims

The overall aim was to provide evidence of the contribution of different prospective risk analysis methods to understanding of risks in healthcare. There were three specific aims of this study. First, we aimed to examine the validity of HFMEA and SWIFT by comparing their outputs in terms of the risks identified and the severity rating of the risks identified. Because risk analysis is a subjective process, there is no criterion against which to assess the validity of the methods. We therefore tested the convergent validity of the methods by investigating the degree of agreement between the two methods in the risks that were identified. Second, we aimed to compare the results of the two prospective methods with the results of prior safety measures, including retrospective risk analysis methods, in order to gain an understanding of the contribution of prospective methods for understanding risk. We compared results with the risks identified by a range of existing activities in a clinical service: namely, root cause analysis, interviews with pharmacists and other staff, and reports of the service’s clinical governance board. Finally, we aimed to elicit healthcare workers’ opinions about the methods, including their views of the benefits and limitations.

## Method

### Design

The study involved comparing the results from three different analyses of the risks present in the service. Two of these, HFMEA and SWIFT, are prospective methods and workshops using these methods were carried out on matched groups of service personnel on the same day as an experimental design. The results were compared with those of prior safety work in the service that had been carried out at various times. These were root cause analyses, analysis of clinical governance board meetings, and a prior interview study.

### Setting

We applied the methods in a community-based anticoagulation service in which pharmacists manage patients receiving anticoagulant medication (warfarin). The service analysed is part of the North Central London Anticoagulation and Stroke Prevention Service (NCLASPS), an established integrated care service including hospital outpatient departments and over 30 community sites. There are several clinical conditions for which treatment or prevention includes giving an anticoagulant. Examples include stroke, mechanical heart valve replacement and venous thrombosis in the leg. The National Audit Office estimated the annual cost of stroke alone in England is about £2.8 billion for direct NHS care and £5.2 billion including informal care [20]. Patients on warfarin require regular monitoring, usually achieved by attending a clinic about every 6-8 weeks, entailing a blood test to determine the blood’s clotting behaviour, expressed through the international normalised ratio (INR). NCLASPS is a successful service: INR control in community patients is good with respect to external standards, and as good as current and historical performance of the local hospital outpatient service.

There have been (and are) various other risk assessment activities in NCLASPS, including an earlier interview study reviewing task analyses, [21] a number of root cause analyses of critical events, and ethnographic work [22]. A clinical governance board meets regularly.

### Participants

There were five participants for each prospective risk analysis method. Different people participated in each of the workshops, but they were matched for disciplinary background and from the same institutions. There were 2 males and 3 females in the HFMEA workshop and one male and four females in the SWIFT workshop. In each workshop, there were 3 pharmacists, 1 administrative assistant and 1 software developer. None of the participants involved had been in a previous task analysis study [21] and none had used PHA methods before.

The pharmacists and administrators were employed by the lead hospital in NCLASPS. The community based clinics are run by pharmacists and use an outreach model. The senior pharmacists had additional roles overseeing and supporting junior staff, training the junior staff and staff employed by other bodies involved in NCLASPS, and developing the service. The administrators had a patient-facing role, but generally did not attend the clinics. The software team were employed by the university and did not have a patient-facing role, but worked closely with pharmacists and administrators to develop and support the software used in the clinics, namely a combined electronic patient record and decision support system [23].

### Procedure

The study was approved by the Education and Management Research Ethics Panel of King’s College London (REP(EM)/09/10-16). All participants were fully informed and consented to take part.

We ran one session for each method with the same facilitators (PL, SD) sequentially on one day: SWIFT first, HFMEA second. By running sessions sequentially, we could ensure no contact between the participants in the two groups and by having two different participant groups, we could eliminate carryover effects. Having the same facilitators for both sessions controlled for any facilitator effects. However, this did mean that there was a potential carryover effect where the facilitators’ experience in the first session affected their actions in the second session, which would make the two methods appear more concordant than they really are. The facilitators were aware of the nature of the study and tried not to let the answers in the first session affect their actions in the second.

Both sessions were carried out in the same room with refreshments provided. The length of the workshops was predetermined to fit into the available time; SWIFT took 2 hours and the HFMEA took 6 hours (including half an hour for lunch). Two of us (HP, JA) were non-participant observers, taking notes through the day. Both sessions were audio recorded. Participants and facilitators completed feedback questionnaires at the end of both sessions.

In both sessions, a hierarchical task analysis diagram (available in [21]) was used to guide the analysis. This was developed in a previous study of the same service, based on interview data, observations and documentary analysis. Although hierarchical task analysis diagrams are not frequently used in healthcare risk assessments or quality improvement work, they have some advantages over the more commonly used sequential diagrams in some situations, including for discussing work problems with a colleague, and for developing a more detailed representation of the work [21]. For these reasons we chose to use hierarchical task analysis diagrams in this study. By having both sessions use this same task analysis, we controlled for any differences in the identification of activity steps.

During the SWIFT workshop, a series of questions was asked at each step of the task analysis designed to probe what would happen if there was deviation from normal practice. For example, step 1.1 of the task analysis is “Obtain patient list for community-based clinic”. The facilitators posed the question “What if the patient list was not obtained”, and followed up with additional questions to probe the expected effect of that failure. The output is a list of risks ranked by severity. At the end of the session, participants were asked, as a group, to select three of the risks identified that they considered to be of particular importance. HFMEA is a well-documented process [6,18] in which a team is assembled to graphically map the process of care, examine it for hazards, determine the severity of the hazards, the likely causes, and recommend actions. After hazards are identified and rated, analysts decide which hazards should proceed to analysis of causes and recommended actions. We followed these steps with some amendments to take into account time constraints and to incorporate the risk matrix commonly used in UK hospitals. As described above, we used an existing hierarchical tasks analysis diagram to guide the analysis. Each step in the task analysis was examined for how it could fail and potential causes of the failure. Failure modes were then rated for likelihood and severity. Likelihood was scored on a five point scale - rare (1), unlikely (2), possible (3), likely (4) and almost certain (5). Severity was scored using a five point scale ranging from none (1), minor (2), moderate (3), major (4) to catastrophic (5). A risk matrix was then used to obtain a risk rating of green, yellow, amber or red. For example, a rare failure with a moderate impact would be rated yellow, while a likely failure with a catastrophic impact would be rated red. Failures rated amber or red were then examined using a decision tree to determine whether further analysis of causes, actions and outcome measures should be undertaken. If so, this is termed proceeding with the analysis.

We also collected data about participants’ opinions of the usefulness of the process and the outcomes. We adapted a questionnaire used in previous studies investigating participants’ experiences of using prospective risk analysis techniques [7]. This contained 15 open-ended questions in three areas: reactions to the workshop process, usefulness of the outcomes, and impact on risk awareness. The qualitative responses to the questions were content analysed and categorised. Response frequencies are reported.

Separate to the planned experiment on prospective methods, prior work by us and the clinical team had been ongoing to identify and ameliorate risks in the service for both clinical and research purposes, [22,23] and indeed continues to this day. This work, involving three main components, was available to us. There had been a series of root cause analyses of critical incidents. These had been led by HP and another researcher and had involved clinical (pharmacist) and administrative members of NCLASPS. Notes from each analysis were circulated. The study that had developed the task analysis also involved additional staff interviews to identify areas of risk [21]. Only the task analysis developed in that study was made available to participants in this study, but these further elements of the earlier study were considered in our analysis of the results. The service has a clinical governance board that meets regularly, a few times a year, to consider a range of issues. Membership of the board includes clinical (pharmacist, medical) and administrative NCLASPS staff, clinical staff from other services, the software team, commissioners, researchers and patient representatives. HP was a participant observer at the board’s meeting and also carried out informal interviews to identify risks. Minutes from all board meetings were available [22].

In order to analyse the degree of overlap of the results of each prospective method, a spreadsheet was prepared listing the risks identified by the HFMEA and SWIFT by section of the task analysis. Two researchers (HP, JA) independently compared the lists of hazards identified by the methods and decided whether there was a match in the other list, a partial match or no substantial match. Disagreements were considered and consensus reached. Comparisons with the prior safety work in the service were done on a narrative basis alone (by HP and JA).

## Results

### Number of risks identified by each prospective method

SWIFT identified 61 risks, which are shown broken down by task analysis section in Table 1, together with the three most important risks identified.

**Table 1:** Number of risks identified by SWIFT

**Task analysis section**	**Total number of risks identified**	**Number of most important risks**
1.1 Obtain patient list for community-based clinic	16	1 (‘Failure or delay in referral’)
1.2 Invite patient	7	0
1.3 Schedule appointment	5	1 (‘Incomplete checklist information - judgement call, treatment delayed, patient does not get treatment’)
2.1 Obtain recent medical history	13	0
2.2 Obtain international normalised ratio (INR)	5	1 (‘Incorrect recording of INR – cannot change INR once entered’)
2.3 Determine dose and time until next appointment	10	0
2.4 Conclude appointment	5	0
**TOTAL**	**61**	**3**

The HFMEA identified 72 top-level risks, rated by participants for severity and likelihood on defined scales, and identified for each whether it would be valuable to proceed with further analysis or not. Table 2 shows the number of risks, the number of risks with a “proceed” decision, and the number of severe risks with a proceed decision, broken down by task analysis section.

**Table 2:** Number of risks identified by HFMEA

**Task analysis section**	**Total number of risks**	**Number of risks with proceed decision**	**Number of risks with proceed decision and a red risk rating**
1.1 Obtain patient list for community-based clinic	8	3	1
1.2 Invite patient	12	4	1
1.3 Schedule appointment	13	9	2
2.1 Obtain recent medical history	14	7	1
2.2 Obtain international normalised ratio	5	2	0
2.3 Determine dose and time until next appointment	8	7	4
2.4 Conclude appointment	12	10	3
**TOTAL**	**72**	**42**	**12**

### Types of risks identified by each prospective method

Two of us (HP, JA) independently compared the two lists and rated risks as matching or not. Our initial judgements showed a kappa of 0.48 (moderate agreement), but both raters also raised the issue of partial matches. We thus decided to add a partial matching category and, through further iterations of the same process and discussion of any disagreements, reached a consensus decision.

An example of a matching risk would be in section 2.1 (obtain recent medical history). The SWIFT identified ‘Fail to identify need for physical examination’ and the HFMEA identified ‘Failure to consider physical examination’. In a few cases, a risk identified by one method was divided into more than one risk by the other. For example, in section 1.2 (invite patient), SWIFT identified ‘Patient is unaware of invite’, while the HFMEA identified two separate risks as ‘Failure for patient to receive invite (hospital to community)’ and ‘Failure for patient to receive invite (community invite)’. Thus, this was marked for the SWIFT as 1 risk also identified by HFMEA, but for the HFMEA as 2 risks also identified by SWIFT.

Partial matches reflected some overlap between risks identified by the two methods. For example, in section 1.2, HFMEA identified ‘Failure to identify/be made aware of special [patient] requirements (physically or mentally)’, while SWIFT identified ‘Unaware of patient transport needs’. Transport needs are one type of special patient requirement, so this was marked as a partial match.

Another example, from section 2.4 (Conclude appointment), illustrates several of these points. The SWIFT identified ‘Failure to insert or incorrect insertion of dose and duration [in the record]’ and ‘Failure to record reason [for dose changes]’, while the HFMEA identified ‘Incorrect documentation’, ‘Incomplete documentation’ and ‘Failure to document’. We concluded these two risks from the SWIFT were covered by the three risks from the HFMEA and so scored them as being matched. We also scored ‘Incorrect documentation’ and ‘Incomplete documentation’ as being matched by the SWIFT risks. However, the HFMEA’s ‘Failure to document’ represented a broader risk than the SWIFT’s more specific ones, so we scored it as being only partially matched. Meanwhile, other risks in this section identified by both methods had no match. For example, the SWIFT identified ‘Fail to put appointment into PAS [patient administration system]’, which had no equivalent in the HFMEA; and the HFMEA identified ‘Failure to ensure patient understands new dose and duration’, which had no equivalent in the SWIFT.

Table 3 summarises the results by section of the task analysis. Each row gives the number of risks identified in that section by the two methods. It then shows how many of those risks were matched, partially matched or not matched by the other method. As described above, matching is not necessarily a 1:1 process. Consider the row for section 1.2 in Table 3. As explained above, there was 1 SWIFT risk that matched a pair of risks in the HFMEA: thus, we see “1 also identified by HFMEA” in the SWIFT column and “2 also identified by SWIFT” in the HFMEA column. There were two pairs where a SWIFT risk partially matched the HFMEA risk, but there was also an additional HFMEA risk (‘Inability to contact patient’) that we decided was partially covered by SWIFT’s ‘Patient is unaware of invite’ (already rated as fully matched). Thus, this produces “2 partially identified by HFMEA” in the SWIFT column, but “3 partially identified by SWIFT” in the HFMEA column. There were then additional risks identified by both methods with little correspondence: thus, “4 not identified by HFMEA” in the SWIFT column and “7 not identified by SWIFT” in the HFMEA column.

**Table 3:** Comparison of HFMEA and SWIFT

**Section**	**Risks identified by SWIFT**		**Risks identified by HFMEA**	
1.1 Obtain patient list for community-based clinic	16 risks identified	3 also identified by HFMEA	8 risks identified	2 also identified by SWIFT
		1 partially identified by HFMEA		1 partially identified by SWIFT
		12 not identified by HFMEA		5 not identified by SWIFT
1.2 Invite patient	7 risks identified	1 also identified by HFMEA	12 risks identified	2 also identified by SWIFT
		2 partially identified by HFMEA		3 partially identified by SWIFT
		4 not identified by HFMEA		7 not identified by SWIFT
1.3 Schedule appointment	5 risks identified	0 also identified by HFMEA	13 risks identified	0 also identified by SWIFT
		1 partially identified by HFMEA		1 partially identified by SWIFT
		4 not identified by HFMEA		12 not identified by SWIFT
2.1 Obtain recent medical history	13 risks identified	6 also identified by HFMEA	14 risks identified	5 also identified by SWIFT
		2 partially identified by HFMEA		4 partially identified by SWIFT
		5 not identified by HFMEA		5 not identified by SWIFT
2.2 Obtain international normalised ratio	5 risks identified	4 also identified by HFMEA	5 risks identified	4 also identified by SWIFT
		1 partially identified by HFMEA		0 partially identified by SWIFT
		0 not identified by HFMEA		1 not identified by SWIFT
2.3 Determine dose and time until next appointment	10 risks identified	1 also identified by HFMEA	8 risks identified	1 also identified by SWIFT
		4 partially identified by HFMEA		4 partially identified by SWIFT
		5 not identified by HFMEA		3 not identified by SWIFT
2.4 Conclude appointment	5 risks identified	2 also identified by HFMEA	12 risks identified	2 also identified by SWIFT
		0 partially identified by HFMEA		1 partially identified by SWIFT
		3 not identified by HFMEA		9 not identified by SWIFT
**TOTAL**	**61 risks identified**	**17 also identified by HFMEA**	**72 risks identified**	**16 also identified by SWIFT**
		**11 partially identified by HFMEA**		**14 partially identified by SWIFT**
		**33 not identified by HFMEA**		**42 not identified by SWIFT**

Overall, although the HFMEA and SWIFT results overlap in many areas, there is substantial non-matching.

For both methods, less than half the hazards were identified by the other method. Both methods highlight more and less serious hazards. The SWIFT picked out three key areas to be addressed. Two of these were identified in the HFMEA, although they were not seen there as high risk: incorrect recording of patient’s INR; and incomplete information in medical history. The third, failure or delay in referral (of a patient into the service), while having no good match in the HFMEA, is a broad concept that covered similar territory to a number of HFMEA-identified hazards.

The HFMEA produced 12 proceed hazards with the highest risk rating: none of these had full matches in the SWIFT, 5 had a partial match (inaccurate patient list; failure to give appropriate treatment between referral into system and invitation to first appointment; failure to insert accurate information into the electronic record; failure to understand the limitations of the decision support algorithm; failure to obtain second opinion when appropriate) and 7 had no match (incorrect updating of computer system; potential to lose non-attenders if not scheduled; failure to receive comprehensible information from physical examination of patient; document incorrect dose; failure to ensure patient understands new dose and duration; failure to have capability to edit incorrect information in the record; failure to give patient correct documentation). There are 30 further proceed hazards: 8 of these had a match in the SWIFT, 3 had a partial match and 19 had no match.

Given two separate attempts to measure risks in the system, under certain assumptions, we can apply a capture-recapture methodology to estimate the total number of risks. For the sake of computation, we count partial matches as half matches. Thus, HFMEA finds 72 risks, including 37% (95% confidence interval: 26-49%) of those identified by SWIFT, and SWIFT captures 61 risks, including 32% (95% confidence interval: 22-43%) of those identified by HFMEA. This implies, using the Chapman method, [24] a total of 190 risks may exist.

### Comparison of prospective methods with prior safety work

The HFMEA and SWIFT differed substantially in terms of hazards recognised compared to prior safety work in the service. Root cause analyses and ethnographic work identified communication between NCLASPS and other healthcare services, particularly GPs, as a key problem area, but these issues were largely absent from the HFMEA or SWIFT results. Inter-service communication issues also featured in the earlier interview study [21], although other findings there fit the SWIFT/HFMEA results better.

Patient understanding has been identified as a central safety issue in prior work: patient understanding was raised in discussion in the HFMEA group, but was ‘parked’ in the discussion as it did not readily fit the structure of the process and was subsequently not included in the final results. It was not recorded by the SWIFT group.

Governance board observations have included considerable discussion over the choice of coagulometer in the clinic and procedures to quality assure the machines. General health and safety issues, *e.g.* the suitability of the physical space for a clinic, have also emerged. These were largely absent in the SWIFT or HFMEA.

Other work has identified potential problems around hygiene and sharps safety. In the context of blood samples being taken, we would normally expect hazards analysis to pay some attention to such issues, but they were never mentioned in either the HFMEA or SWIFT. It is also notable that no mention was made of possible mismatches between the electronic record and the patient’s personally-held paper record.

### Participants’ feedback

The acceptability of both methods was high and participants viewed the process as positive (Table 4). The meetings were good-tempered, constructive and enjoyed, with all participants contributing. Participants thought that the hazards identified by both methods were serious and realistic, but only HFMEA participants thought that it identified new hazards and HFMEA participants were more likely than SWIFT participants to state that the workshop had increased their understanding of risk in the service. All participants thought that the workshops were easy to understand and that team participation in the discussion was high. Notably, nine of the ten participants thought that the process required more time. Feedback from the facilitators indicated that the workshops were interactive and participative.

**Table 4:** Participant opinions of the workshops

**Questions**	**HFMEA**	**SWIFT**
In your opinion did the workshop identify the most serious hazards in the care of patients?	Yes 4	Yes 5
	No 1	No 0
Did the workshop identify realistic hazards? That is, things that realistically might go wrong?	Yes 5	Yes 5
	No 0	No 0
Did the workshop identify new hazards that you were not aware of before?	Yes 4	Yes 5
	No 1	No 0
Has the workshop changed your understanding of the risks in your work?	Yes 4	Yes 1
	No 1	No 4
Was the process easy to understand?	Yes 5	Yes 5
	No 0	No 0
Did everyone contribute to the discussion?	Yes 5	Yes 5
	No 0	No 0
What were the main benefits of the workshop? (free text; multiple answers possible)	• Identified hazards (4)	• Discussion (2)
	• Identified actions (1)	• Increased understanding of the clinic (1)
	• Increased awareness of risks (1)	• Forum for reflection (1)
	• Increased understanding of the clinic (1)	
	• Systematic (1)	
What were the main limitations of the workshop? (free text; multiple answers possible)	• Lack of time (5)	• Lack of time (4)
	• Session was long (1)	
	• Dry (1)	

## Discussion

The aim of this study was to investigate the validity of HFMEA and SWIFT by comparing the results of these two methods, and by comparing the workshop results with risk data from other activities. Both prospective methods seek to comprehensively identify the significant risks in a service: if they achieve that goal, they should produce similar results. This is a test of convergent validity. We have no ‘gold standard’ answer as to what the main risks in the system are, but if the results of the two prospective methods differ substantially, we can safely conclude that at least one of the methods is not valid. Other safety work in the NCLASPS service – including root cause analyses, an interview study [21] and clinical governance board discussions – provides additional reference points.

Although there was some overlap between the two PHA methods, each identified many unique risks not identified by the other. The substantial number of non-matches demonstrates, as with other research, [16,17,25] that these prospective techniques cannot be relied upon to be comprehensive. They find many significant hazards, but do not find all potential hazards. Healthcare services should not be complacent and presume that a single HFMEA or SWIFT is sufficient to ensure all risks have been identified.

The fact that the two methods produced different results may not be surprising considering the SWIFT workshop lasted for two hours compared with six for the HFMEA. The time difference can be partly accounted for by the fact that HFMEA analyses risks and identifies controls that can reduce the risk, whereas SWIFT only analyses risks. One of our motivations for the study was to establish whether more streamlined methods such as SWIFT are as useful as those requiring more time, given time may be one of the barriers to the use of PHA methods in healthcare [9,17]. The HFMEA workshop identified more risks and more serious risks than SWIFT, and this was confirmed by participant feedback that SWIFT did not identify new hazards or increase understanding of risks in the system. On this basis, SWIFT would not appear to be as effective as HFMEA for analysing clinical processes for risks. Nevertheless, SWIFT identified hazards that were not identified by HFMEA, suggesting that it could be useful in some circumstances or used adjunctively with another method [8].

Both methods rely on subjective judgements made by participants and, thus, differences between the two methods may well stem from the different participants in our two groups. However, these methods are designed to largely overcome such subjectivity through their structured and systematic approach. The expectation should be that different participants will produce largely similar results.

Not only do the SWIFT and HFMEA show poor concordance with each other, but they showed even poorer concordance with a multitude of other data sources about hazards in the service, as seen in other work [25]. A central problem here is that of scope. Both SWIFT and HFMEA are based on a task analysis that defines the scope of the subsequent PHA. The task analysis diagram that we used was based on those elements of the service directly under the service’s control and did not include processes beyond this boundary. Root cause analyses and interviews, however, strongly indicated that the most serious risks lie when patients cross organisational boundaries between services. There is nothing inherent in task analysis or PHA methods that excludes inter-organisational work and careful scoping of the analysis at an early stage might suggest that an inter-organisational risk analysis is the best strategy. We conclude it is important to bear in mind how decisions on scope at an early stage of the work (here, the task analysis) can have effects down the line, and to consider questions of scope carefully and flexibly. Choices over scope should be justified with respect to other sources of evidence (*e.g.* retrospective analyses). We suggest that clinical and research teams need to act with fuzzy boundaries: that is, one should not reject issues as being out of the scope of the analysis – rather, these need to be recorded and reviewed regularly to see whether the scope needs to change.

SWIFT and HFMEA are based on task analyses or process maps, which analyse processes as a sequential series of acts. However, many aspects of a clinical service are not easily represented in a process map. In this study, patient education, the suitability of the premises and the correct functioning of the coagulometer were omitted from the process map and from the SWIFT and HFMEA analyses. A cognitive work analysis framework based on functions that need to be fulfilled, rather than task steps to be completed, may be more useful [26].

This study had several limitations. The number of participants was small. The size of the service meant that only two groups of 5 participants each were eligible to take part. Different results may have been obtained if the groups were bigger or if several workshops were conducted for each method. This was not possible in the setting in which the study was conducted, but this reflects the real constraints and dilemmas faced by small clinical groups in applying these methods. Likewise, while our facilitators were experienced, our participants were not familiar with these methods beforehand, yet these are the sorts of healthcare professionals that are being encouraged to adopt PHA techniques.

The comparative design required that both workshops take place on the same day to ensure that participants did not discuss the workshops with each other and therefore bias the results. This meant that we used a briefer HFMEA procedure than is often recommended. While this does reflect the real pressures in clinical contexts on professionals’ time, a longer HFMEA may have captured more risks. The use of a more thorough HFMEA is congruent with our recommendations and the views of the workshop participants. We were able to omit the step of graphically mapping the process by using a process map that had been developed in a previous study, allowing us to save time and provide an equal comparison with the SWIFT. However, we note that our findings are, thus, on the process of risk assessment and do not cover that part of the HFMEA method that involves mapping the process of care (in contrast to [18]) or identifying risk control interventions.

Third, we used the same facilitators for both sessions to control for any effects caused by facilitator experience and style. However, this opened the possibility of a carryover effect, with what the facilitators did in the second session being influenced by what they had heard in the first session. The facilitators were aware of the experimental design and sought to counter any such effect. If there were any carryover effect, it would decrease any differences between the two techniques. Given that we observed many differences between the two methods, we argue that this has not affected the results greatly.

## Conclusions

The results showed that there were many differences in the outputs produced from these two PHA methods. Both methods identified important hazards and were judged as useful and positive by the participants. The qualitative nature of the methods means that the results could be expected to vary between methods and with different participants. However, the collaborative discussion of risks by a healthcare team is powerful and it may be the case that raising awareness of risks in the system through these techniques is as important a factor in increasing safety as the identification of hazards. There is a danger that the structured nature of the methods leads practitioners to treat the outputs as highly reliable and valid, but our results and those of others [11,18,25] show that the results should be viewed as part of an emerging picture of risk built from a variety of sources.

A clinical service seeking to ensure high quality should use a range of different risk management methods and not assume any one approach is comprehensive. It remains an open question how much risk analysis is enough, but we suggest that multiple prospective and multiple retrospective methods should be considered and data from different analyses integrated to provide a comprehensive view [11]. In particular, issues of scope need careful consideration throughout the process. We recommend that these PHA techniques should not be used in isolation, but should be one tool among many within an ongoing safety strategy.

## Competing interests

The authors declare that they have no competing interests.

## Authors’ contributions

HP: conceived the study, developed the method, organised the setting, conducted data collection, analysed the results, wrote the first draft. JA: conceived the study, developed the method, conducted data collection, analysed the results, contributed to the final draft. LC: conceived the study, developed the method, developed the task analyses, contributed to the final draft. PL, SD: developed the method, facilitated the sessions, conducted data collection. JB: developed the method. All authors read and approved the final manuscript.

## Pre-publication history

The pre-publication history for this paper can be accessed here:

http://www.biomedcentral.com/1472-6963/14/41/prepub
